# Vanillic acid and vitamin C attenuated di-2-ethylhexyl phthalate-induced testicular toxicity in adult male rats

**DOI:** 10.1530/RAF-22-0045

**Published:** 2022-09-26

**Authors:** B Ogunlade, S C Gbotolorun, O A Adedotun, K Iteire, J Adejayi

**Affiliations:** 1 Behavioral and Aging Lab, Human Anatomy Department, Federal University of Technology Akure, Ondo State, Nigeria; 2 Anatomy Department, University of Lagos, Lagos, Nigeria; 3 Anatomy Department, University of Medical Sciences, Ondo City, Ondo State, Nigeria

**Keywords:** di-2-ethylhexyl phthalate, vanillic acid, vitamin C, testicular toxicity, antioxidant, histology

## Abstract

**Abstract:**

Di-2-ethylhexyl phthalate (DEHP) is an extensively used plasticizer which has raised some concerns about its safety on human health. This study aimed at evaluating the effects of vanillic acid (VA) and vitamin C (VC) supplementation on DEHP-induced testicular toxicity. Thirty-five adult male Wistar rats were randomly divided into 7 groups (A–G) (*n = 5*) receiving distilled water; 250 mg/kg bw of DEHP only; 30 mg/kg bw of VA and 250 mg/kg bw of DEHP; 30 mg/kg bw of VC and 250 mg/kg bw of DEHP; 30 mg/kg bw of DEHP plus 30 mg/kg bw of VA and 30 mg/kg bw of VC; 30 mg/kg bw of VA only; and 30 mg/kg bw of VC only, respectively. At the end of the experiment, blood was taken from the heart via cardiac puncture and stored, semen was collected from the caudal epididymis for immediate sperm analysis, while the testes were excised and preserved for histological examination and biochemical analysis. The results showed a significant decrease (*P* < 0.05) in body weights, sperm motility, sperm volume, sperm viability and count, antioxidant levels, and reproductive hormonal levels, with a significant increase (*P* < 0.05) in sperm morphological defect and lipid peroxidation level in DEHP-only group compared with the control but was ameliorated after VA and VC administration compared to the DEHP-only treated animals. VA and VC supplementation attenuated the toxic effects of DEHP on the testicular functions, morphology, and semen characterization of the experimental adult male Wistar rats.

**Lay summary:**

Male infertility is considered when identifiable female causes of infertility are excluded and semen quantity and quality fail to fulfil World Health Organization criteria. From conception through to adulthood, people are exposed to limitless environmental toxicants among which di-2-ethylhexyl phthalate (DEHP) commonly found in personal care products, cosmetics, and medical devices is prevalent. The present study elaborated on the importance of taking antioxidant-rich foods containing vitamin C and vanillic acid, such as those found in various fruits, olives, whole wheat, and cereal grains, in combating infertility caused by environmental toxicants. An experiment was carried out on rats to see the effect of vanillic acid and vitamin C supplementation on preventing DEHP-induced testicular toxicity. The testicles and semen were analyzed from five rats in each treated and control groups. The data led us to conclude that vanillic acid and vitamin C supplementation do have attenuating effects on DEHP-induced testicular toxicity, due to their high antioxidant and anti-inflammatory properties.

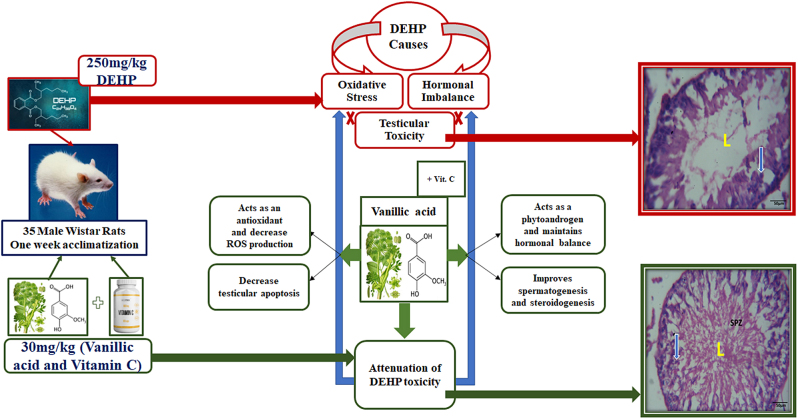

## Introduction

Infertility in males refers to a man’s failure to achieve conception in a fertile female (Olooto 2012). Male infertility is frequently due to sperm and semen quality defects (Cooper *et al.* 2010). It is estimated that 60% of married couples having regular unprotected intercourse achieve pregnancy after 6 months of co-habitation, 90% achieve pregnancy by 12 months, and 95% between 18 and 24 months (Agboola 2005).

Evidence has shown that endocrine-disrupting chemicals (EDCs) have probable deleterious consequences on development, growth, metabolism, and reproduction, as they can intervene in the production, release, transport, metabolism, binding movement, or removal of the natural hormones inside the body (Nassouri *et al.* 2012). Majority of the previous findings on the endocrine disruptors categorized as anti-androgens deal with phthalates (Albert & Jegou 2014). Phthalic esters are compounds widely used as plasticizers. Worldwide, nearly three million tons of phthalic esters are produced per year, and they can be found in many everyday products, including plastic bags, polyvinylchlorides (PVCs), cosmetics, food packaging, industrial paints, and also in blood transfusion packs (Muczynski *et al.* 2012). Because of the non-covalent nature of their link with plastics, phthalates can leach from these products easily and, therefore, be ingested (Kavlock *et al.* 2002). Di-2-ethylhexyl phthalate (DEHP) is the most common member of the class of phthalates. DEHP is also called bis(2-ethylhexyl) phthalate or dioctyl phthalate (Huang *et al.* 2008). From conception through to adulthood, people are exposed to limitless anthropogenic and naturally occurring EDCs, among which DEHP is the most extensively used plasticizer in PVC plastics, which is prevalent in personal care products, cosmetics, and medical devices, accounting for ~80% of the total phthalates’ global consumption (Kamrin 2009). As DEHP is not chemically bound to PVC, it leaches easily, migrates, and evaporates into indoor air and the surrounding atmosphere, foodstuffs, and other materials (Zhang *et al.* 2018). DEHP is a well-known reproductive toxicant. Extensive studies on phthalate-induced male reproductive toxicity have been reported since Shaffer *et al*. (1945) published the first report on DEHP-induced testicular injury in an animal model. The mechanism by which DEHP causes testicular toxicity has not been fully elucidated (Erkekoglu *et al.* 2010). Previous evidence showed that DEHP via activation of peroxisome proliferator-activated receptors exerts its anti-androgen effect by inhibiting fetal testosterone biosynthesis (Culty *et al.* 2008), and subsequent suppression of antioxidant enzymes, leading to the production of free radicals and oxidative stress, which contributes to oxidative DNA damage (O’Brien *et al.* 2005).

Antioxidants when applied to foods of humans and animals minimize the rate of chemical compound’s toxicity (Ansar 2013). They prevent toxicity by stimulating the body’s detoxifying enzymes or by inhibiting the ultimate production of carcinogenic metabolites (Ansar 2013). Rich sources of diversified pharmacological products have been found to be fruits, spices, vitamins, and even various herbs (Ansar & Iqbal 2013). Such reagents have antioxidant and free-radical scavenging properties (Wang *et al.* 2012, Zeng *et al.* 2013). Vanillic acid (VA) is a phenolic derivative from edible plants and fruits known to possess antimicrobial, anti-filarial (Varma *et al.* 2013), and antibacterial effects (Rai *et al.* 2006). It may be found in a variety of fruits, olives, whole wheat, and cereal grains, as well as in wine, beer, and cider (Siriamornpun & Kaewseejan 2017). The main phenolic components in samples of potatoes (*Solanum tuberosum* L.) were identified by Kim *et al.* (2019). The scientific name of VA is 4-hydroxy-3-methoxy benzoic acid (Zeng *et al.* 2013). It is an oxidized portion of vanillin (4-hydroxy-3 methoxybenzaldehyde) (Sinha *et al.* 2008), exhibiting several bioactive properties, including antimicrobial action against yeasts, moulds (Tipparaju *et al.* 2005), and bacteria (Rupasinghe *et al.* 2006) and as an antioxidant (Burri *et al.* 2009). Vanillin has antimutagenic, anticlastogenic, and antitumor properties and may hence be known as a nutraceutical agent (Shyamala *et al.* 2007). VA also functions as an intermediate in the ferulic acid synthesis of vanillin (Cinolani *et al.* 2010). The antioxidant activity of VA seems to be an important action (Wang *et al.* 2012). The *in vitro* antioxidant mechanisms of VA include free radical scavenging activity, reducing power, and inhibition of lipid peroxidation (Chou *et al.* 2010). Furthermore, VA reduced lipid peroxidation products and significantly restored enzymatic antioxidants and nonenzymatic antioxidants in the plasma of hypertensive rats (Kumar *et al.* 2011). In view of the facts mentioned earlier, this study is focused on the antioxidant effect of VA and vitamin C (VC) supplementation on DEHP-induced testicular toxicity in adult male Wistar rats.

## Materials and methods

### Chemicals

DEHP was procured from Sigma Company, and VA and VC were procured from Max International, Salt Lake, UT, USA. All other chemicals used in the study were of analytical-reagent grade.

### Animals

In this prospective cohort study, a total of 35 adult male Wistar rats weighing 150–200 g and aged 8–10 weeks (*Rattus norvegicus*) were obtained from the animal house, Department of Human Anatomy, Federal University of Technology, Akure. The rats were collected in isolated cages in the experimental house of the Department of Human Anatomy, College of Health Science, Federal University of Technology, Akure. They were maintained with a constant 12 h light/12 h darkness cycle. All animal handling procedures were approved by the Ethics Committee of the Federal University of Technology, Akure CHS/FUA/2021/031.

### Experimental protocol

The rats were divided into seven groups (*n* = 5), labeled as groups A, B, C, D, E, F, and G. DEHP dosage was administered accordingly using the protocol by Zhang *et al.* (2018), while VA and VC dosage were administered accordingly using the procedure by Ma *et al.* (2020).

- Group A represents control and received water as placebo(negative control).
- Group B received 250 mg/kg bw of DEHP only (positive control).
- Group C received 30 mg/kg bw of VA and 250 mg/kg bw of DEHP.
- Group D received 30 mg/kg bw of VC and 250 mg/kg bw of DEHP.
- Group E received 250 mg/kg bw of DEHP plus 30 mg/kg bw of VA and 30 mg/kg bw of VC.
- Group F received 30 mg/kg bw of VA only.
- Group G received 30 mg/kg bw of VC only.

All administrations were done orally, co-treatment via gastric gavage daily for 28 days, and all animals were observed for any behavioral anomalies, illnesses, and physical anomalies. The experimental procedures were in accordance with the provided recommendations in the ‘Guide for the Care and Use of Laboratory Animals’ prepared by the National Academy of Sciences. The rats were fed with standard rat chow and drinking water was supplied *ad libitum*. The weights of the animals were recorded at procurement, during acclimatization, at the commencement of the experiment, and weekly throughout the experimental period using a CAMRY electronic scale (EK5055, Indian).

### Surgical procedure

After the last administration, the rats were administered i.p. pentobarbital sodium (40 mg/kg), and their abdominal region was opened and the testes of all the animals were immediately removed. The testicular weights of each rat were recorded. The rats were decapitated and blood samples were collected for analysis. The blood samples were centrifuged at 4°C for 10 min at 250 *g*, and the serum obtained was stored at −20°C until assayed. The harvested testis specimens were fixed in Bouin’s fluid for histological analysis (Avwioro 2010).

### Epididymis sperm count, viability, and motility

The spermatozoa from the cauda epididymis were obtained by cutting into 2 mL of medium (Hams F10) containing 0.5% BSA (Feng *et al.* 2001). After 5 min of incubation at 37°C (with 5% CO_2_), the cauda epididymis sperm reserves were determined using a hemocytometer. Sperm motility was analyzed with a microscope (Leica DM750) and reported as the mean number of motile sperm according to the method developed by the World Health Organization (WHO 1999).

### Biochemical estimations

The level of lipid peroxidation products was estimated in accordance with the method published by Niehaus and Samuelsson (1968). Nonenzymatic antioxidants such as reduced glutathione (GSH) and enzymatic antioxidant markers such as catalase (CAT) were estimated as described by Ellman (1959) and Sinha (1972), respectively; superoxide dismutase (SOD) activity in the testes was determined according to the method described by Marklund and Marklund (1974).

### Hormone determination

The hormonal levels of testosterone (TT), follicle-stimulating hormone (FSH), and leutenizing hormone (LH) were measured using available immunoassay (ELISA) kits (Randox Laboratories Ltd, Admore Diamond Road, Crumlin, Co., Antrim, United Kingdom, Qt94QY) according to manufacturer’s instructions.

### Testicular histology preparation

The testes of the rats were harvested and fixed in Bouin’s fluid for 24 h before being transferred to 70% alcohol for dehydration. The tissues were passed through 90% and absolute alcohol and xylene for different durations before being transferred into molten paraffin wax for 1 h each in an oven at 65°C for infiltration. The tissues were embedded, and serial sections cut on a rotary microtome set at 5 μm were performed. The tissues were picked up with albumenized slides and allowed to dry on hot plates for 2 min. The slides were dewaxed with xylene and passed through absolute alcohol (two changes), 70% alcohol, 50% alcohol, (in that order), and then in water for 5 min. The slides were then stained with hematoxylin and eosin, mounted in DPX, and photomicrographs were taken at a magnification of 100× on a Leica DM750 microscope (Adelakun *et al.* 2019).

### Statistical analysis

The data obtained were analyzed statistically using one-way ANOVA, followed by Dunnett’s comparison test. Data were expressed as mean ± s.e.m. The level of significance was at *P* < 0.05. Data were analyzed using GraphPad Prism 5 Windows (GraphPad Software).

## Results

### Effect of VA and VC supplements on the body weight on DEHP induced normal and experimental rats

**Figure 1:**
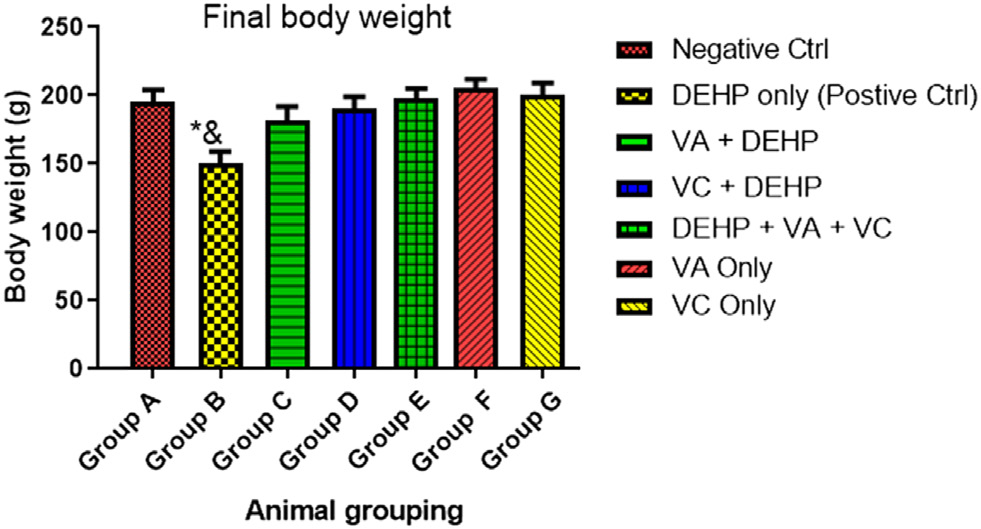
Effect of VA and VC supplements on the body weight on DEHP-induced normal and experimental rats. *P < 0.05 as compared to group A; ^&^*P* < 0.05 as compared to groups C, D, E, F, and G.

The result revealed that rats treated with DEHP only (group B) showed significant decrease (*P* < 0.05) in body weight when compared with the control (group A) (Fig. 1). There was also a significant difference in the body weights of the animals treated with DEHP only (group B) when compared with the animals administered with VA and DEHP (group C), VC and DEHP (group D), DEHP plus VA plus VC (group E), VA only (group F), and VC only (group G) groups (Fig. 1).

### Effect of VA and VC supplements on testis weight (right and left) on DEHP-induced normal and experimental rats

**Figure 2:**
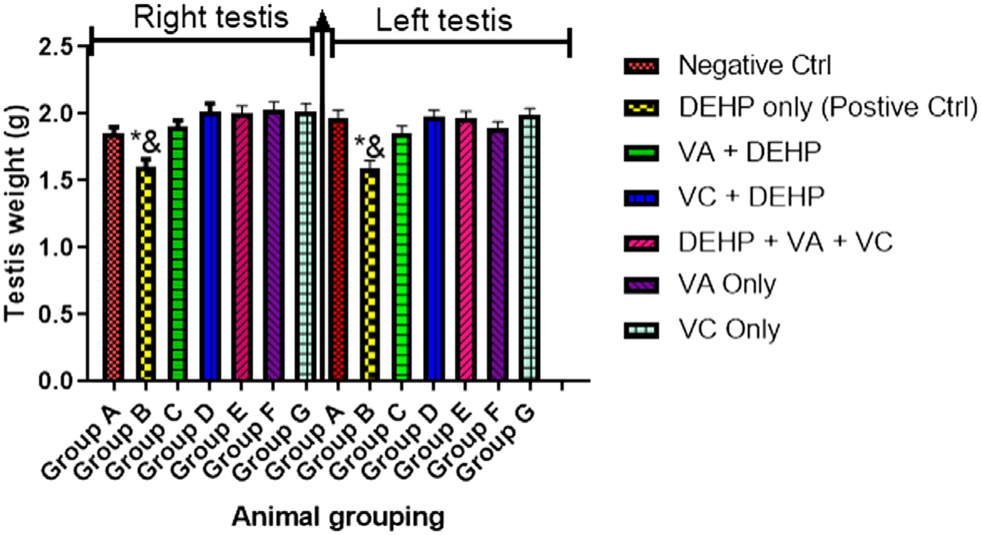
Effect of VA and VC supplements on testis weight (right and left) on DEHP-induced normal and experimental rats. *P < 0.05 as compared to group A; ^&^*P* < 0.05 as compared to groups C, D, E, F, and G.

The result revealed that rats treated with DEHP only (group B) showed a significant decrease (*P* < 0.05) in testis weight (both left and right) when compared with the control (group A) (Fig. 2). There was also a significant difference in testis weight (both left and right) of the animals treated with DEHP only (group B) when compared with the animals administered with VA and DEHP (group C), VC and DEHP (group D), DEHP plus VA plus VC (group E), VA only (group F), and VC only (group G) groups (Fig. 2).

### Effect of VA and VC supplements on sperm morphology (neck, tail, and head defects and normal) on DEHP-induced normal and experimental rats

**Figure 3:**
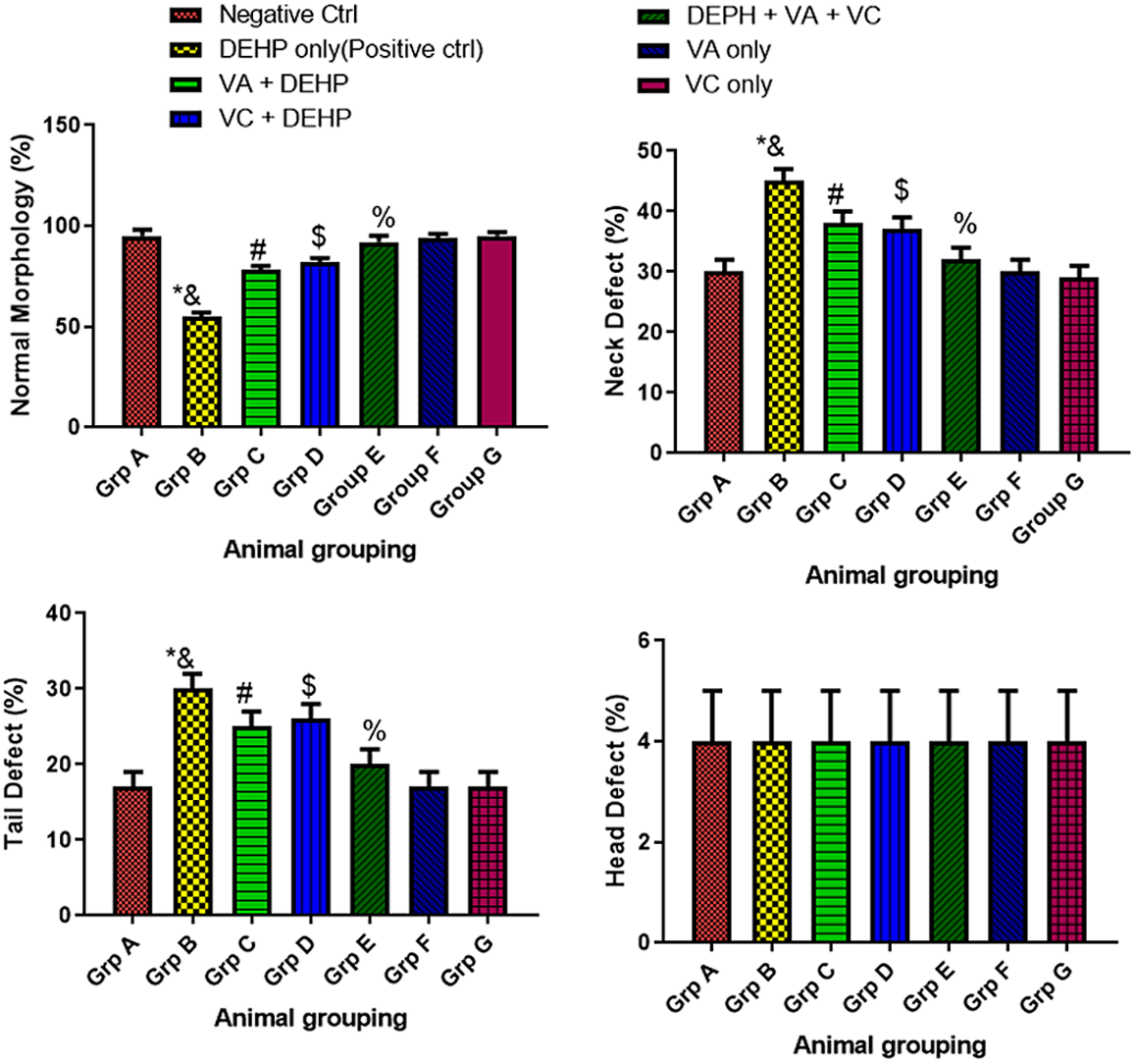
Effect of VA and VC supplements on sperm morphology (neck, tail, and head defects and normal) on DEHP-induced normal and experimental rats. *P < 0.05 as compared to group A; ^&^*P* < 0.05 as compared to group E; ^#^*P* < 0.05 as compared to group B; ^$^*P* < 0.05 as compared to group B; ^%^*P* < 0.05 as compared to group A.

The result revealed that rats treated with DEHP only (group B) showed a significant increase (*P* < 0.05) in defective sperm morphology (neck defect (ND) and tail defect (TD)), but no significant difference in head defect (HD) when compared to the control (Fig. 3). However, the rats administered with DEHP plus VA plus VC (group E) showed a significant increase (*P* < 0.05) in sperm morphology (normal) and a decrease in sperm morphological defects (ND, TD), but no significant difference in HD when compared with the DEHP only group (group B) (Fig. 3). Furthermore, the animals treated with VA plus DEHP (group C) and also VC plus DEHP (group D) showed a significant improvement (*P* < 0.05) in sperm morphology (normal) and a decline in sperm morphological defects (ND, TD), but no significant difference in HD when compared with the DEHP only group (group B) (Fig. 3). In addition, there was no significant difference in the levels of sperm morphology (normal) and sperm morphological defects (ND, TD, and HD) in the animals treated with VA only and VC only (group F and G) when compared with the control (Fig. 3).

### Effect of VA and VC supplements on sperm motility, concentration count, semen volume, and sperm viability on DEHP-induced normal and experimental rats

**Figure 4:**
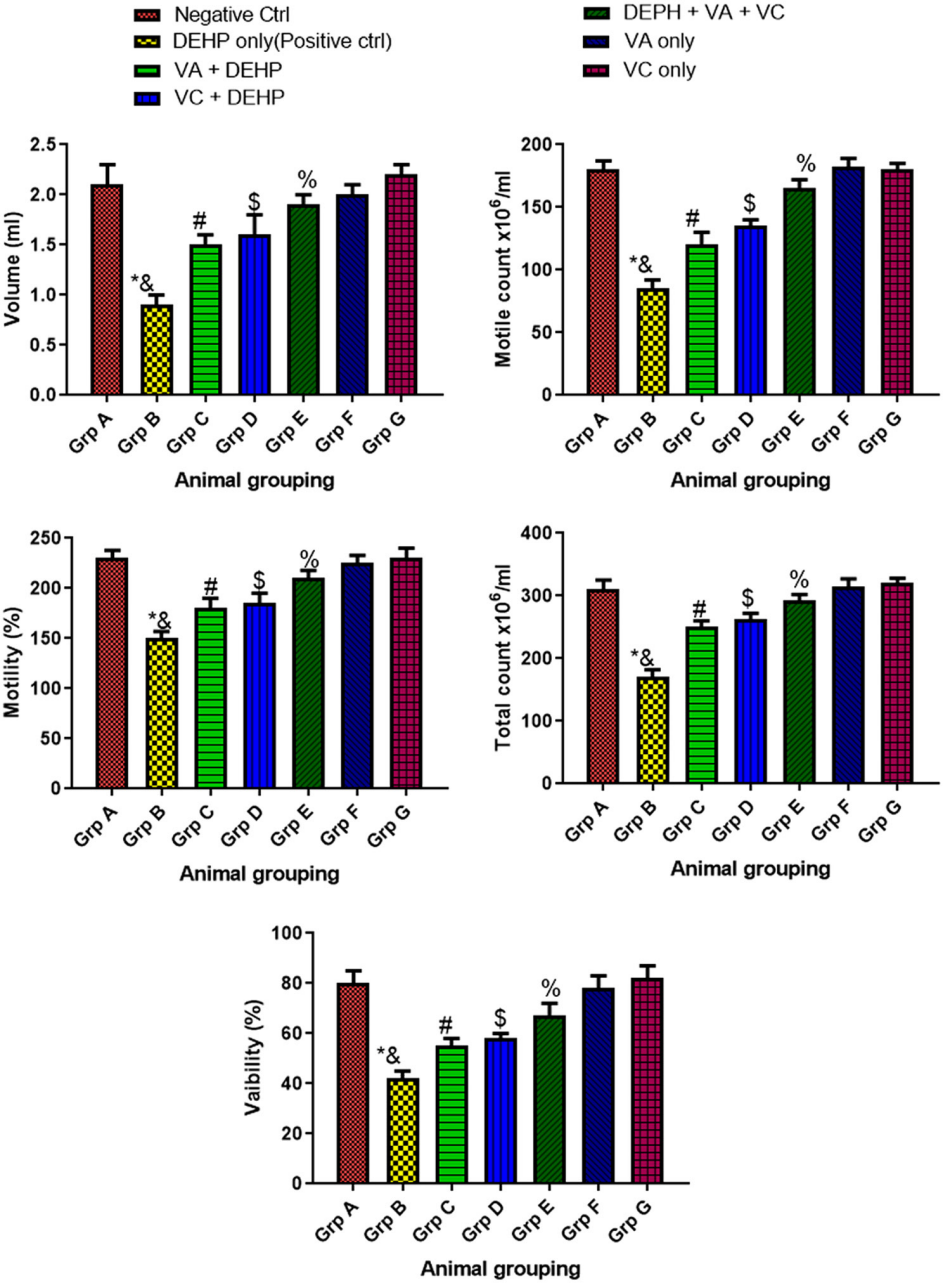
Effect of VA and VC supplements on sperm motility, concentration count, semen volume, and sperm viability on DEHP-induced normal and experimental rats. *P < 0.05 as compared to group A; ^&^*P* < 0.05 as compared to group E; ^#^*P* < 0.05 as compared to group B; ^$^*P* < 0.05 as compared to group B; ^%^*P* < 0.05 as compared to group A.

The results revealed that rats treated with DEHP only (group B) showed a significant decline (*P* < 0.05) in sperm motility, concentration count, semen volume, and sperm viability relative to the control group (Fig. 4). However, the rats administered with DEHP, VA, and VC (group E) showed a significant increase (*P* < 0.05) in sperm motility, concentration count, semen volume, and sperm viability when compared with the DEHP only group (Fig. 4). In addition, there was a significant decrease (*P* < 0.05) in these characteristics when the rats administered with DEHP only was compared with the rats treated with VA plus DEHP (group C) and VC plus DEHP (Fig. 4). Furthermore, there was no significant difference between the control group and animals administered with the DEHP, VA, and VC (group E) when compared with each another. There was no significant difference in sperm motility, concentration count, semen volume, and sperm viability when the control (group A) and the animals that received VA only (group F) and VC only (group G) were compared with one another (Fig. 4).

### Effect of VA and VC supplements on MDA, GSH, SOD, and CAT levels on DEHP-induced normal and experimental rats

**Figure 5:**
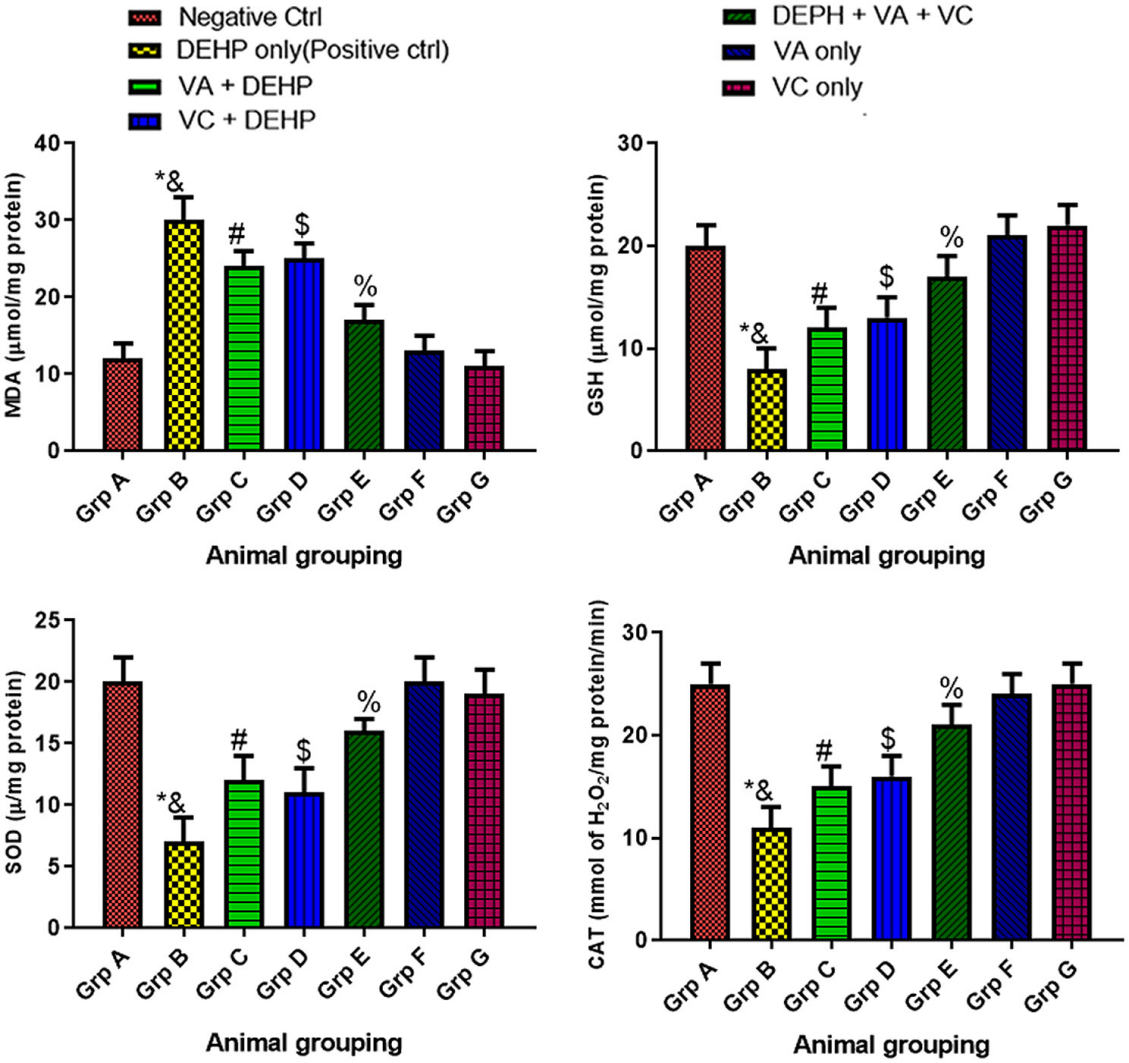
Effect of VA and VC supplements on MDA, GSH, SOD, and CAT levels on DEHP-induced normal and experimental rats. *P < 0.05 as compared to group A; ^&^*P* < 0.05 as compared to group E; ^#^*P* < 0.05 as compared to group B; ^$^*P* < 0.05 as compared to group B; ^%^*P* < 0.05 as compared to group A.

The results revealed a significant increase (*P* < 0.05) in malondialdehyde (MDA) level and a corresponding increase (*P* < 0.05) in CAT, SOD, and GSH levels among the animals treated with DEHP only (group B) when compared to the control group (group A) (Fig. 5). Although, there was a significant increase (*P* < 0.05) in CAT, SOD, GSH levels and a corresponding decrease (*P* < 0.05) in MDA level among the animals that received the combined administration of DEHP plus VA plus VC (group E) when compared with animals treated with DEHP only (group B) (Fig. 5). Furthermore, there was a significant increase (*P* < 0.05) in CAT, SOD, GSH serum levels and a decrease (*P* < 0.05) in MDA serum level among the animals that received VA plus DEHP (group C) and VC plus DEHP (group D) when compared with the DEHP only group (group B) (Fig. 5). In addition, there was a significant increase (*P* < 0.05) in MDA level and a corresponding increase (*P* < 0.05) in CAT, SOD, and GSH levels among the animals that received the combined administration of DEHP plus VA plus VC (group E) when compared to the control group (group A) (Fig. 5). However, there was no significant difference in CAT, SOD, GSH, and MDA levels when the control (group A) and the animals that received VA only (group F) and VC only (group G) were compared with one another (Fig. 5).

### Effect of VA and VC supplements on serum level of FSH, LH, and TT on DEHP-induced normal and experimental rats

**Figure 6:**
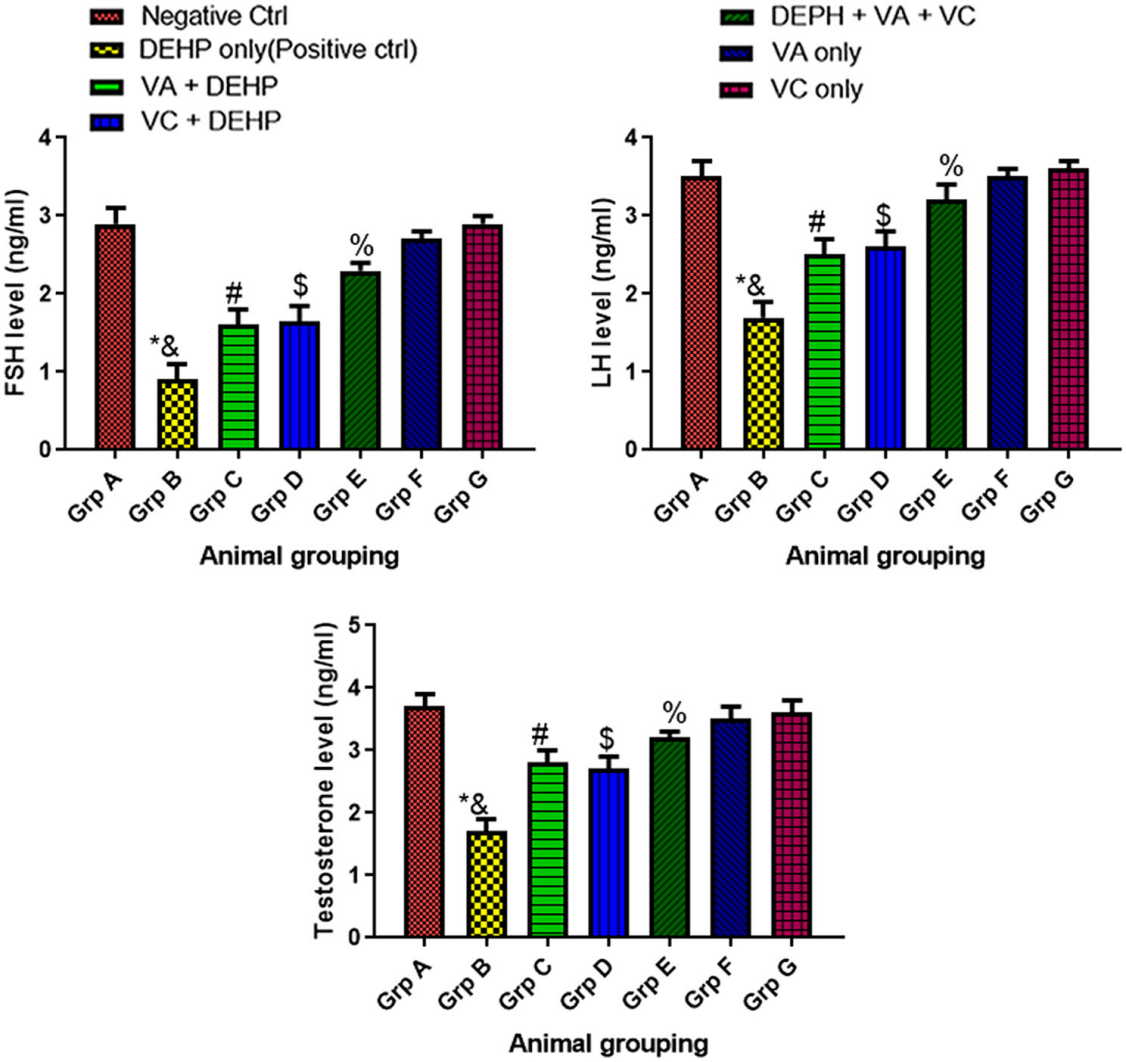
Effect of VA and VC supplements on serum level of FSH, LH, and TT on DEHP-induced normal and experimental rats. *P < 0.05 as compared to group A; &: *P* < 0.05 as compared to group E; ^#^*P* < 0.05 as compared to group B; ^$^*P* < 0.05 as compared to group B; ^%^*P* < 0.05 as compared to group A.

There was a significant decrease (*P* < 0.05) in FSH, LH, and TT serum levels in animals that were treated with DEHP only (group B) when compared to the control group (group A) (Fig. 6). However, there was a significant increase (*P* < 0.05) in FSH, LH, and TT serum levels among the animals that received of DEHP plus VA plus VC (group E) when compared with animals treated with DEHP only (group B) (Fig. 6). In addition, there was a significant increase (*P* < 0.05) in FSH, LH, and TT serum levels among the animals that received VA plus DEHP (group C) and VC plus DEHP (group D) when compared with the DEHP only group (group B) (Fig. 6). Although, there was no significant difference in FSH, LH, and TT serum levels when the control (group A) and the animals that received DEHP plus VA plus VC (group E), VA only (group F) and VC only (group G) were compared with one another (Fig. 6).

### Testicular photomicrographs showing the effect of VA and VC supplements on DEHP-induced normal and experimental rats

**Figure 7:**
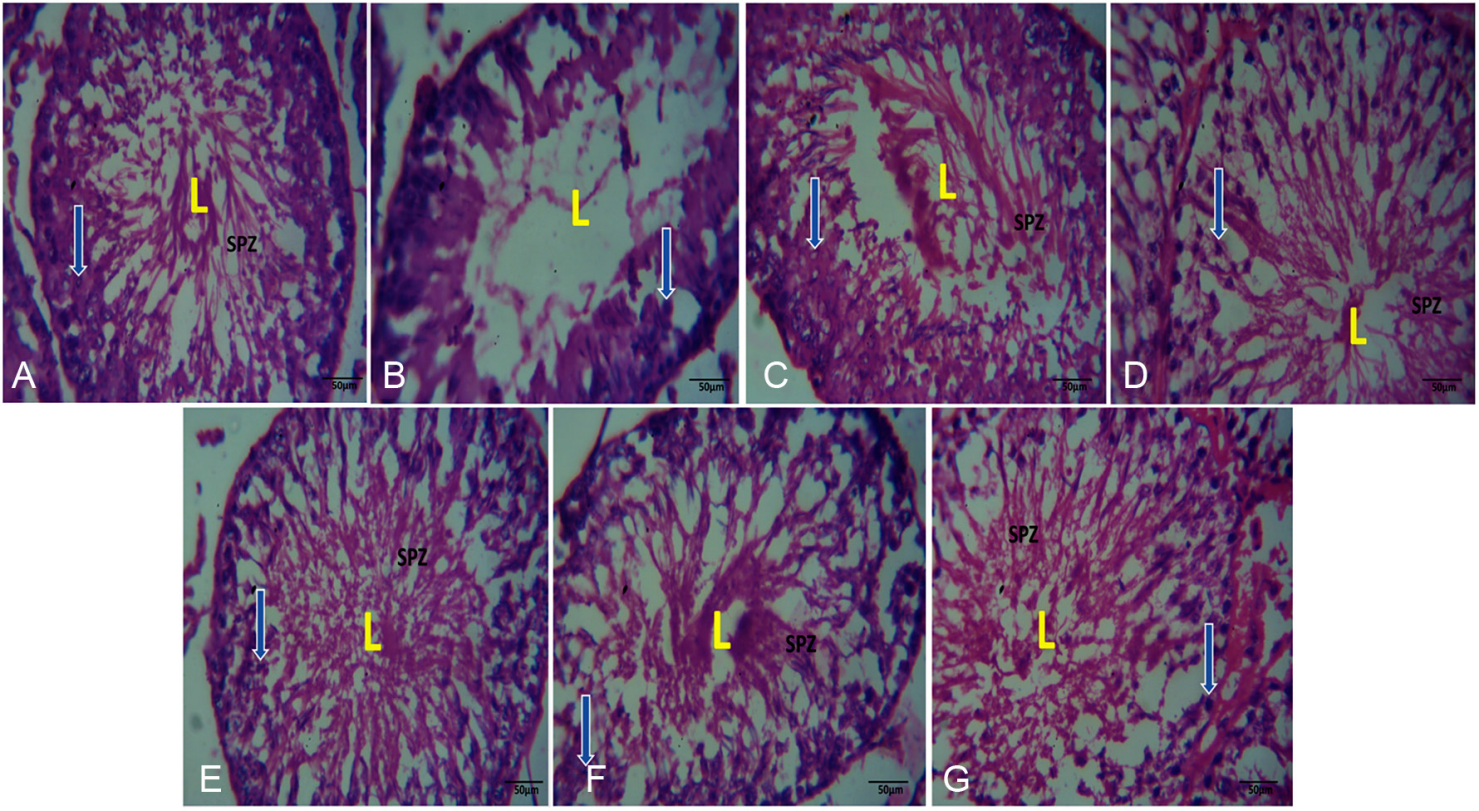
(A) Group A: testicular photomicrograph of the control group; (B) Group B: testicular photomicrograph of the DEHP-only treated group; (C) Group C: testicular photomicrograph of the vanillic acid plus DEHP treated group; (D) Group D: testicular photomicrograph of the vitamin C plus DEHP treated group; (E) Group E: testicular photomicrograph of the DEHP plus vanillic acid and vitamin C treated group; (F) Group F: testicular photomicrograph of the vanillic acid-only treated group; (G) Group G: testicular photomicrograph of the vitamin C only treated group. L, lumen; SPZ, spermatozoa; arrow, spermatogenic cells.

The testicular histoarchitecture of the DEHP only group (B) (Fig. 7B) showed necrosis and degeneration with a decrease in germinal epithelium thickness and a reduction in the diameter of the seminiferous tubules when compared with the control (group A) (Fig. 7A). In addition, DEHP caused distortion in the seminiferous tubules with loss of normal distribution of epithelial lining and vacuolar cytoplasm compared with the control. However, testicular photomicrograph of the control group had similar characteristics with the VA only (group F) and VC only (group G) treated animals, showing oval or circular presentation with distinctive stratified seminiferous epithelium whose lumen possesses spermatogenic cells and prominent Leydig cells (Fig. 7F and G). The testicular section of the groups administered with both DEHP, VA, and VC (group E) as well as those administered with DEHP and VA (group C) and DEHP and VC (group D) showed restored microarchitecture of the testicular morphology showing mild distortion of the tubular architecture and disorganization of the spermatogenic cells in seminiferous tubules (Fig. 7C, D and E).

## Discussion

Human male infertility is a worldwide condition that affects around 7% of all males and accounts for 40–50% of all cases of infertility (Jungwirth *et al.* 2012). The testis is vulnerable to a number of stressors, including heat, inflammation, radiation, and exposure to substances that cause germ cell apoptosis (Angulo *et al.* 2011). Lifestyle factors, as well as various environmental agents, may impair male fertility (Pflieger-Bruss *et al.* 2004). Male gonadal function is affected by a large number of factors including age, smoking, alcohol consumption, environmental agents, and occupational factors (Latini *et al.* 2006). Male subfertility is closely related to environmental endocrine disruptors (Chevrier *et al.* 2012, Nordkap *et al.* 2012). One of the most representative environmental endocrine disruptors is DEHP, which has been shown to cause abnormal male reproductive development possibly through an oxidative stress-related mechanism (Tetz *et al.* 2013, Skinner 2016). DEHP is rapidly hydrolyzed to mono(2-ethylhexyl)-phthalate (MEHP) when it enters the body. Subsequently, MEHP inhibits mitochondrial respiration and generates excessive reactive oxygen species (ROS), such as H_2_O_2_ (Tang *et al.* 2018). These actions increase the oxidative stress level, which can lead to DNA damage, and eventually causes cell death or apoptosis (Tetz *et al.* 2013). Because oxidative stress is one of the primary causes of germ cell apoptosis, the testis contains high levels of antioxidants including ascorbic acid, often known as VC, to protect germ cells against oxidative damage (Liang *et al.* 2012). VC deficiency actually causes disturbance of spermatogenesis; thus, the defense mechanism against oxidative stress plays a critical role in the maintenance of spermatogenesis (Angulo *et al.* 2011). Natural compounds are always a prime choice for the treatment of various diseases. Various researchers explore natural compounds in* in vitro* and* in vivo* studies. VA is a phenolic molecule that is an oxidized molecule of vanillin. VA may be found in a variety of foods, including fruits, whole grains, juices, herbs, beers, green tea, and wines (Sharma *et al.* 2020). It has been shown to have anti-inflammatory, antioxidant, cardioprotective, immunostimulating, hepatoprotective, neuroprotective, and antiapoptotic properties (Kim *et al.* 2011, Vinothiya & Ashokkumar 2017). The present study evaluated the antioxidative effect of VA and VC supplementation on DEHP-induced testicular toxicity in mice.

The result from this study revealed that rats treated with DEHP only showed a significant decrease in body weight when compared with the control group. Body weight is a good indicator of the negative effects of xenobiotics, and it is considered a determinant parameter of toxicity testing (Zahra *et al.* 2020). The reduction in body weight by DEHP is consistent with a study conducted by Abd-Ellah *et al*. (2016). However, there was a significant increase in the body weights of the animals treated with DEHP, VA, and VC when compared with the DEHP-only treated group. The increase in body weight following DEHP administration by VA can be likened to its anti-inflammatory property and analgesic action which is further explained by Calixto-Campos *et al*. (2015).

Apoptosis is a process of cell death that occurs not only in normal germ cells but also in cells damaged by toxic substances (Park *et al.* 2002). From this study, it was also discovered that rats treated with DEHP only showed a significant increase in defective sperm morphology when compared to the control. DEHP can be said to have produced a marked increase in apoptosis in testis. DEHP’s testicular toxicity at gonadotoxic levels lowers litter size in male rats, which is linked to reduced epididymal sperm density, testicular atrophy, and an increase in abnormal sperm numbers (Agarwal *et al.* 1986). However, on co-administration of DEHP, VA, and VC, there was a significant increase in normal sperm morphology and a corresponding decrease in sperm morphological defects when compared with the DEHP-only treated group. This result is in accordance with Oyagbemi *et al*. (2016).

The testicular sperm counts, volume, motility, and viability are important indicators of spermatogenesis (Wang *et al.* 2014). In this study, the administration of DEHP only reduced sperm motility, concentration count, sperm volume, and viability when compared with the control. These results come in accordance with Ma *et al*. (2012), who showed an obvious reduction in the total sperm count, volume, motility, and viability after DEHP treatment. However, on co-administration of DEHP, VA, and VC, there was a significant increase in sperm motility, concentration count, sperm volume, and viability when compared with the DEHP-only treated group. This result is consistent with the outcome of a study carried out by Calixto-Campos *et al*. (2015).

Antioxidants are vital in the defense mechanism against pathological events, but the imbalance of free radicals and antioxidants may harm the organs and their normal functions (Zahra *et al.* 2020). In most mammalian cells, mitochondria are an important source of ROS which can damage proteins, lipids, and DNA (Poprac *et al.* 2017). The first line of defense against ROS in the body is endogenous enzymes system such as SOD, CAT, and GSH while MDA is a marker for oxidative stress. The results of this study revealed a significant increase in MDA level and a corresponding increase in CAT, SOD, and GSH levels among the animals treated with DEHP only. This is in accordance with a study conducted by Tetz *et al*. (2013) which stated that DEHP exposure leads to the production of excessive ROS. Antioxidants fight against free radicals and serve as protection against a variety of ailments. The hunt for effective free radical scavenger and gonadoprotective sources has been a constant pursuit. From this study, the co-administration of DEHP, VA, and VC significantly increased the CAT, SOD, and GSH levels while reducing the MDA level. The scavenging effects of VA might be attributed to the presence of polyphenolics, carotenoids, and other antioxidant constituents. This supports earlier research on the antioxidant and anti-inflammatory properties of VA (Kumar *et al.* 2011, Bezerra *et al.* 2016).

The testis is an androgen-dependent organ and reproductive hormones such as the LH, FSH, and TT are essential to maintain the structure and function of the testis and accessory sex glands (Rezvanfar *et al.* 2013). From this study, there was a significant decrease in FSH, LH, and TT serum levels in animals that were treated with DEHP only when compared to the control group. The mechanisms by which DEHP causes its toxic effects on the reproductive system, according to Ge *et al*. (2007) and Noriega *et al*. (2009), are related to its anti-androgenic potential. Leydig cells are the primary source of TT and other major reproductive hormone production in males, and differentiation of Leydig cells in the testes is one of the primary events in the development of the male body and fertility (Zhang *et al.* 2008). Available data suggest that Leydig cells are one of the main targets of phthalates (Ge *et al.* 2007). However, on co-administration of DEHP, VA, and VC, there was a significant increase in FSH, LH, and TT serum levels when compared with animals treated with DEHP only. This is consistent with previous studies by Hashem *et al*. (2018) and Sharma *et al*. (2020).

The histopathological findings from this study showed necrosis and degeneration with a decrease in germinal epithelium thickness and a reduction in the diameter of the seminiferous tubules in animals administered with DEHP only when compared with the control group. In addition, DEHP also caused a distortion in the seminiferous tubules with a loss of normal distribution of epithelial lining and vacuolar cytoplasm when compared with the control. These findings are in accordance with a study conducted by Gangolli (1982) who reported that the testicular photomicrographs of DEHP-treated rats showed a marked reduction in the diameter of seminiferous tubules, the germinal epithelium consisted only of Sertoli cells, spermatogonia and a few spermatocytes, and also a cessation of spermatogenesis. However, restored microarchitecture of the testicular morphology showing mild distortion of the tubular architecture and disorganization of the spermatogenic cells in seminiferous tubules was observed in animals administered with DEHP, VA, and VC. This can be attributed to the anti-inflammatory and antiapoptotic coupled with the antioxidative properties of VA (Agarwal *et al.* 1986, Bezerra *et al*. 2016) as well as the ability of VC in maintaining spermatogenesis (Angulo *et al.* 2011).

## Conclusion

It can be concluded from this study that DEHP (a well-known plasticizer and an environmental toxicant) is detrimental to male gonads and can lead to testicular toxicity. However, VA (a phenolic-rich substance) together with VC supplementation showed great potential in attenuating the toxic effects of the phthalate in question, most especially its toxic effects on the testis.

## Declaration of interest

The authors declare that there is no conflict of interest that could be perceived as prejudicing the impartiality of the research reported.

## Funding

This work did not receive any specific grant from any funding agency in the public, commercial, or not-for-profit sector.

## Ethics approval

The experimental procedures were conducted in accordance with the NIH guidelines for the care and use of laboratory animals in line with guidelines of the Department of Human Anatomy, College of Health Science, Federal University of Technology, Akure and the Health Research and Ethics Committee of the Federal University of Technology, Akure CHS/FUA/2021/031.

## Author contribution statement

B Ogunlade: conceptualization, methodology, validation, writing-review and editing, investigation. S C Gbotolorun: methodology, project administration, supervision, investigation. O A Adedotun: formal analysis, investigation, writing-original draft. K Iteire: writing-original draft, investigation. J Adejayi: writing-original draft, proof-reading and formatting.
